# The Role of Rituximab in the Treatment of Primary Central Nervous System Lymphoma

**DOI:** 10.3390/cancers13081920

**Published:** 2021-04-16

**Authors:** Ruben Van Dijck, Jeanette K. Doorduijn, Jacoline E.C. Bromberg

**Affiliations:** 1 Department of Hematology, Erasmus MC Cancer Institute, University Medical Center Rotterdam, 3015 GD Rotterdam, The Netherlands; r.vandijck@erasmusmc.nl (R.V.D.); j.doorduijn@erasmusmc.nl (J.K.D.); 2 Department of Neuro-Oncology, Erasmus MC Cancer Institute, University Medical Center Rotterdam, 3015 GD Rotterdam, The Netherlands

**Keywords:** primary central nervous system lymphoma (PCNSL), non-Hodgkin lymphoma, rituximab, high-dose methotrexate

## Abstract

**Simple Summary:**

Primary central nervous system lymphoma (PCNSL) is a rare form of cancer and the treatment of newly diagnosed patients is challenging. Many chemotherapy regimens are being used, and methotrexate is an important component in most. The role of the immunotherapy rituximab is not as clear. This review focuses on the available evidence for the use of this monoclonal antibody in the treatment of patients with PCNSL.

**Abstract:**

Primary central nervous system lymphoma (PCNSL) is a type of non-Hodgkin lymphoma limited to the central nervous system. It has a poor prognosis. Consensus has been reached on the treatment of newly diagnosed patients with high-dose methotrexate-based chemotherapy, but whether the addition of the monoclonal anti-CD20 antibody rituximab improves survival, as it does in systemic B-cell non-Hodgkin lymphoma, remains disputed. In this review, we reflect on the available evidence of the use of rituximab in PCNSL. Whether rituximab has any beneficial effect remains uncertain.

## 1. Introduction

Primary central nervous system lymphoma (PCNSL) is a non-Hodgkin lymphoma limited to the central nervous system (brain, leptomeninges, spinal cord and eyes) [1]. It is a unique World Health Organization (WHO) hematological entity and a rare form of cancer, accounting for 3% of all brain malignancies, with an age-standardized incidence of 0.4–0.5 per 100,000 per year [2,3]. The International Extranodal Lymphoma Study Group (IELSG) identified the following independent predictors of poor prognosis in PCNSL: age above 60 years, Eastern Cooperative Oncology Group (ECOG) performance status greater than 1, elevated serum lactate dehydrogenase (LDH) level, elevated cerebral spinal fluid protein concentration, and the involvement of deep regions of the brain. Prognosis can be estimated depending on the number of risk factors involved, with 2-year survival estimates of 80%, 48%, and 15% in patients with 0–1 factors, 2–3 factors and 4–5 factors, respectively [4]. A similar validated prognostic model from the Memorial Sloan-Kettering Cancer Center identifies three prognostic classes with significantly distinguished outcome: class 1 consisting of patients <50 years, class 2 including patients > or =50 years and Karnofsky performance score (KPS) > or =70 and class 3 concerning patients > or =50 years and KPS < 70 [5]. The overall incidence of PCNSL is increasing, especially in the elderly [6,7,8]. The standard-of-care treatment for patients with PCNSL consists of induction chemotherapy, usually a high-dose methotrexate-based regimen because of high CNS-bioavailability (related to blood–brain barrier permeability), and consolidation therapy with whole-brain radiotherapy or autologous hematopoietic stem cell transplantation if feasible [9,10,11,12,13,14]. This treatment has improved prognosis, but patient outcomes remain poor as compared to other forms of extranodal diffuse large B-cell lymphoma [15,16]. There is no consensus on the approach for relapsed PCNSL disease [17,18]. 

In the treatment of systemic B-cell non-Hodgkin lymphoma, the introduction of the chimeric monoclonal antibody rituximab, targeting the cell surface molecule CD20, has improved outcomes considerably, and the combination of chemo- and immunotherapy has become the standard of care [19,20,21]. The value of this therapy in PCNSL, however, remains uncertain, and the scarcely available evidence is not definitive. One of the main concerns of such use is whether intravenously administered rituximab, a relatively large molecule, reaches sufficient levels in the central nervous system to exert adequate efficacy. A disrupted blood–brain barrier secondary to lymphoma infiltration might theoretically be more permeable [22,23], and together with its proven beneficial effect in systemic B-cell non-Hodgkin lymphoma, might constitute improved outcomes in rituximab-treated PCNSL patients. Here, we summarize and reflect on the available evidence related to the use of rituximab in the treatment of PCNSL. 

## 2. Materials and Methods

**Figure 1:**
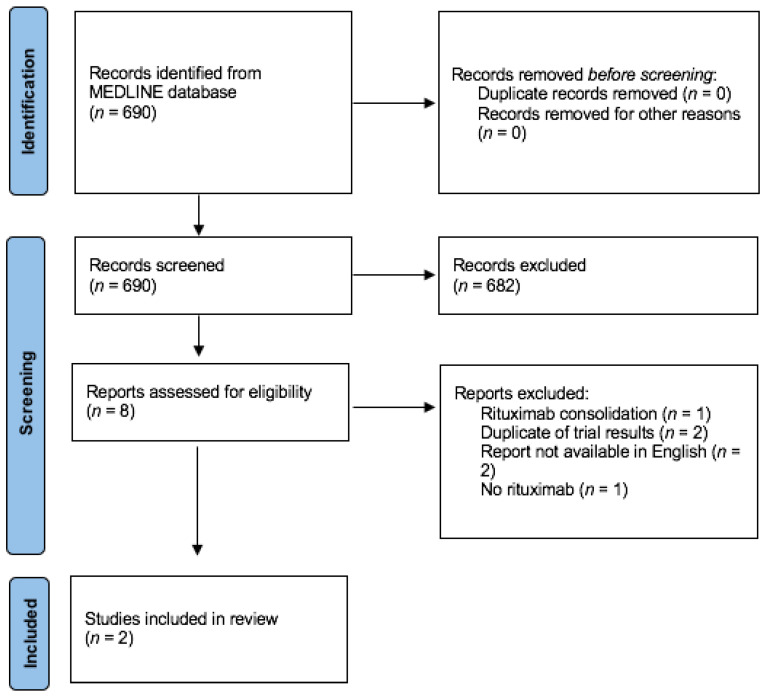
Flow diagram of systematic review according to Preferred Reporting Items for Systematic Reviews and Meta-Analyses (PRISMA) guidelines.

We searched the MEDLINE database for data published on this subject between the initial US Food and Drug Administration approval of rituximab on 26 November 1997 and 1 March 2021, with the following Medical Subject Headings (MeSH) search terms: “Lymphoma, Non-Hodgkin” (MeSH) or “non-Hodgkin lymphoma” (all fields) and “Central Nervous System” (Mesh) or “central nervous system” (all fields) and “primary central nervous system lymphoma”(all fields) or “PCNSL”(all fields) and “Rituximab” (MeSH) or “rituximab”(all fields) and “Randomized Controlled Trial” (publication type). Criteria for inclusion in systematic analysis were all randomized controlled trials that compared outcomes between a rituximab-treated group and a group receiving the same treatment without rituximab in newly diagnosed PCNSL patients (= or >18 years of age) without a history of systemic lymphoma or prior treatment for PCNSL. The diagnosis must be neuropathologically confirmed. The main criteria for exclusion were studies that included patients with human immunodeficiency virus seropositivity or other forms of immunodeficiency, the presence of systemic lymphoma and prior treatment for PCNSL or refractory disease where rituximab was not given as first-line treatment. No eligibility restrictions based on performance status were considered. Outcomes of interest were overall survival, defined as the time from randomization to death due to any cause, and progression-free survival, defined as time from randomization to the progression of lymphoma, or event-free survival, defined as the time from randomization to a complication or event that treatment was intended to prevent. Only papers with an abstract written in English were taken into consideration. This query resulted in 690 records, which were screened on title and abstract, and after the exclusion of 682 records for obvious ineligibility (main causes for exclusion at this point were: no mention of PCNSL, no use of rituximab, presence of systemic lymphoma, and no randomized controlled trial), the remaining 8 reports were assessed for eligibility. Six of these were deemed ineligible due to reasons mentioned in Figure 1. Ultimately, two randomized controlled trials in which newly diagnosed patients with PCNSL and no history of systemic lymphoma were randomized in first-line treatment between a rituximab-containing chemotherapy group and a control group given the same treatment without rituximab, which were eligible for further analysis. These two randomized controlled trials (IELSG32-trial and HOVON/ALLG-trial) are compared in Table 1. The meta-analysis of this evidence and multiple reviews of the literature are available and discussed below. 

## 3. Review of the Literature 

### 3.1. Randomized Controlled Trials 

The first randomized controlled trial on this subject was published in the “Lancet Hematology” by members of the International Extranodal Lymphoma Study Group (IELSG) in 2016 [24]. It was a multi-center randomized phase II trial (IELSG32), in which 227 HIV-negative immunocompetent patients aged 18–70 years with newly diagnosed PCNSL and measurable disease were included between February 2010 and August 2014. These subjects were randomized to receive four courses of methotrexate 3.5 g/m^2^ on day 1 plus cytarabine 2 g/m^2^ twice daily on days 2 and 3 (group A); or the same combination plus two doses of rituximab 375 mg/m^2^ on days –5 and 0 (group B); or the same methotrexate–cytarabine–rituximab combination plus thiotepa 30 mg/m^2^ on day 4 (group C), with the three groups repeating treatment every three weeks. The choice of consolidation therapy in the case of responsive disease was a result of second randomization: whole-brain radiotherapy or autologous hematopoietic stem cell transplantation. The primary endpoint of the first randomization was the complete response (CR) rate after the induction of chemo(immuno)therapy; the secondary endpoint was progression-free survival. When focusing on the effect of rituximab as systemic treatment for PCNSL by comparing the standard-of-care chemotherapy (group A) and standard-of-care chemotherapy plus rituximab (group B), no statistically significant difference in CR rate (HR 0.74, 95% CI 0.43–1.29, *p* = 0.29) can be discerned. At a median follow-up of 30 months, however, the difference in overall response rate (complete response and partial response) between these groups is statistically significant (HR 0.69, 95% CI 0.54–0.88, *p* = 0.010). While results for 2-year progression-free survival show a strong trend toward favoring rituximab treatment, they were not statistically significant, but only by a slight margin (HR 0.52, 95% CI 0.32–0.86, *p* = 0.051). The difference in overall survival was not statistically significant (HR 0.63, 95% CI 0.42–1.02, *p* = 0.095). The combination of methotrexate, cytarabine, rituximab and thiotepa (group C), known as the MATRix-protocol, was associated with a significantly better CR-rate, progression-free and overall survival, as compared to standard-of-care methotrexate-base chemotherapy. Interestingly, the authors note that the reported CR rate (primary endpoint) and overall survival in the methotrexate–cytarabine group was unusually poor, compared to results from the IELSG20 trial (CR rate 23% as compared to 46% in the IELSG20 trial, which is similar to the reported 49% CR rate in the MATRix-group), making it a poor comparator arm [25]. In conclusion, the results of this study show that, in the treatment of PCNSL, the addition of rituximab to methotrexate-based chemotherapy is not associated with better CR-rates nor with better progression-free and overall survival. 

The second randomized controlled trial investigating the effect of rituximab in the treatment of PCNSL was published in “Lancet Oncology” in 2019 [26]. It was a multicenter randomized phase III trial (HOVON 105/ALLG NHL 24) including, in the period between August 2010 and May 2016, 200 non-immunocompromised patients with newly diagnosed PCNSL aged 18 to 70 years. The subjects were randomly assigned to receive methotrexate-based chemotherapy with or without rituximab, each 28-day cycle consisting of intravenous methotrexate 3 g per m^2^ on days 1 and 15; intravenous carmustine 100 mg per m^2^ on day 4; intravenous teniposide 100 mg per m^2^ on days 2 and 3; and oral prednisone 60 mg per m^2^ on days 1–5 (the methotrexate, carmustine, teniposide, prednisone (MBVP) combination); with or without intravenous rituximab 375 mg per m^2^ on days 0, 7, 14, and 21 in cycle one and days 0 and 14 in cycle two. Consolidation therapy in case of responsive disease consisted of high-dose cytarabine (all patients) and whole-brain radiotherapy only in younger patients (up until the age of 60 years), with the risk of radiation-induced neurotoxicity taken into account. The primary endpoint in this study was event-free survival, with an event defined as the absence of complete response or unconfirmed complete response at the end of all protocol treatment, or relapse or death after previous complete response or unconfirmed complete response. Secondary endpoints included overall survival, toxicity and the proportion of patients achieving a response after induction or consolidation chemotherapy and after completion of radiotherapy. Event-free survival at 1 year was 49% (95% CI 39–58) in the MBVP group and 52% (42–61) in the rituximab-treated group (HR 1.00, 95% CI 0.70–1.43, *p* = 0.99), showing no added benefit of rituximab to standard chemotherapy with methotrexate, carmustine, teniposide and prednisone. There was also no statistically significant difference in secondary endpoints between both groups. Overall survival at 1, 2, and 3 years was 79% (95% CI 69–86), 65% (55–74), and 61% (51–71), respectively, for the MBVP group, and 79% (69–85), 71% (60–79), and 58% (46–68), respectively, for the R-MBVP group (HR 0.93, 95% CI 0.59–1.44, *p* = 0.74). The authors note similar toxicity between both groups. It is noteworthy that in an unplanned subgroup analysis, however, a possible beneficial effect was found in younger patients (those receiving consolidation with whole-brain radiotherapy up to 60 years of age) as compared to the older ones, with a better median event-free and progression-free survival favoring the rituximab-treated group in the former. In this group, median event-free survival was 19.7 months (95% CI 6.5–not reached) for the MBVP group and 59.9 months (41.4–not reached) for the R-MBVP group (HR 0.56, 95% CI 0.31–1.01, *p* = 0.054). On the other hand, patients older than 60 years had a median event-free survival of 8.3 months (95% CI 4.2–24.8) for the MBVP group and 4.2 months (3.5–10.5) for the R-MBVP group (HR 1.42, 95% CI 0.90–2.23, *p* = 0.13). A similar difference between both groups was noted for progression-free survival. However, there was no significant difference in overall survival. Lower than expected event-free survival was attributed to the high median age of 61 years as compared to other studies. The authors concluded that they have not proven a beneficial effect of adding rituximab to the chemotherapy treatment of newly diagnosed PCNSL patients. 

Further data from two randomized controlled trials from China are available, however, given that their manuscripts were written in Mandarin and only the abstracts were in English, we were not able to evaluate them on the quality of their study protocol, and thus we will not include them in further discussion. The first group included 100 patients with PCNSL diagnosed in one center and randomly assigned to a targeted treatment group (high-dose methotrexate and rituximab) or a control treatment group of high-dose methotrexate and whole-brain radiotherapy. Among the patients in the rituximab-treated group, 66% reached complete remission, as compared to 58% of patients in the comparator arm, with a median progression-free survival of 28 months in the former group and 11 months in the latter [27]. The second study, also a single-center trial, included 58 patients and randomly assigned them to the same treatment groups; high-dose methotrexate and rituximab versus high-dose methotrexate and whole-brain radiotherapy. They mention a significant difference in response rates and survival rates, favoring the rituximab-treated group, with one- and three-year survival rates in the rituximab-treated group of 86% and 62%, respectively, as compared to 58% and 31% in the control group [28]. Both studies mention rituximab as a valuable treatment option in PCNSL patients, but due to their study design, a pure rituximab effect cannot be distilled from these results. 

### 3.2. Data from Cohort Analyses

Data from historical and prospective cohort analyses show a potential beneficial effect of adding rituximab to the systemic treatment of PCNSL, although being only speculative in the absence of a randomized control group [29,30,31,32,33,34,35,36]. A non-exhaustive overview was given in Table 2. This overview is not a result of a systematic analysis of the literature. As with all cohort analysis, these results are not validated with a randomized control group and clinicians must be prevented from drawing major conclusions on the efficacy of rituximab in PCNSL treatment from these data. They can, however, show that combination treatment with chemotherapy and rituximab is at least feasible [29,30,31,32,33,34,35,36,37,38] and not related to major differences in toxicity.

## 4. Discussion

The role of the anti-CD20 monoclonal antibody rituximab in the first-line treatment of PCNSL remains uncertain [39] and the aforementioned evidence cannot support a definitive conclusion. Questions have been raised whether the large molecule rituximab could sufficiently penetrate the blood–brain barrier and exert a therapeutic effect on the central nervous system. It was postulated that the efficacy of intravenously administered rituximab in PCNSL was thus limited by the blood–brain barrier, with the cerebrospinal fluid concentration reaching only 0.1% of serum concentration after intravenous administration [40]. Several preclinical studies have thus investigated the potential use of intrathecal administration of rituximab and other antibodies to bypass the limiting effect of the blood–brain barrier [41,42,43,44] and hypothesized the possibility of a beneficial immunotherapeutic effect within the neuroaxis [45]. Limited data, however, suggest that the blood–brain barrier in patients with leptomeningeal disease secondary to lymphoma infiltration is, at least temporarily, disrupted, allowing for higher cerebral spinal fluid concentrations of systemically introduced rituximab [35]. This hypothesis, together with its proven beneficial effect in systemic B-cell non-Hodgkin lymphoma, was the starting point for further research. Retrospective analyses had already been suggestive of improved survival in rituximab-treated PCNSL patients, as mentioned above. To this date, however, only two randomized controlled trials, the IELSG32-trial and the HOVON/ALLG-trial (Hemato-Oncology foundation for adults in the Netherlands/Australasian leukaemia and lymphoma group), have prospectively investigated the role of rituximab in newly diagnosed PCNSL patients. The IELSG32-trial showed no beneficial effect on CR rates, progression-free and overall survival by solely adding rituximab to a high-dose methotrexate-based chemotherapy regimen, although the difference in progression-free survival showed a trend of borderline significance. The HOVON-105 trial also failed to show an added benefit of rituximab relating to event-free and overall survival in PCNSL treatment. 

A systematic review and meta-analysis of these data was published by Schmitt and colleagues in “Hematological Oncology” in 2019 [46]. Their pooled analysis of the two randomized controlled trials mentioned above (IELSG32 and HOVON/ALLG) concluded that rituximab-treated PCNSL patients showed a better progression-free survival but no benefit in overall survival as compared to standard methotrexate-based chemotherapy-treated patients (for progression-free survival HR 0.65, 95% CI 0.45–0.95; for overall survival HR 0.76, 95% CI 0.52–1.12). These results indicate a possible benefit of rituximab with low certainty. Furthermore, the use of rituximab did not result in more grade 3 or 4 toxicity nor any surplus treatment-related mortality, but neither study in their primary publication mentions any data on quality of life. Their results are further nuanced by mentioning the low certainty of evidence due to imprecise estimates, unexplained heterogeneity, a poorly preforming comparator arm in the IELSG32-trial, and a risk of bias in the assessment of progression-free survival (endpoints in both studies were dependent on MRI interpretation). In another meta-analysis, by Song and colleagues, who included retrospective data, the use of rituximab was closely correlated with a higher 5-year progression-free (OR 2.54, 95% CI 1.64–3.93, *p* < 0.0001) and 5-year overall survival (OR 2.87, 95% CI 2.02–4.08, *p* < 0.00001) [47]. However, the inclusion of retrospective data, with its associated publication bias and other risks of biases, makes this a less reliable estimate. 

The subgroup analysis in the HOVON-trial, suggesting a beneficial effect on event-free survival in younger rituximab-treated patients (up to the age of 60 years), concerned only small groups (47 patients in each arm) and this was an unplanned post hoc analysis. All these patients were consolidated with whole-brain radiotherapy, and during this period received no rituximab. However, rituximab serum levels are known to persist for longer periods of time [48]. It is then postulated by the authors that the surplus event-free survival effect in this group might be related to a renewed uptake of residual systemic rituximab in the central nervous system, when the blood–brain barrier is again disrupted secondary to whole-brain radiotherapy. This, however, remains conjecture, and no grand conclusions can be drawn from such a post hoc analysis. Follow-up data concerning overall survival in this study could possibly show whether the improvement in event-free survival in this subgroup also results in a difference in overall survival. There is a need for future randomized studies powered to evaluate specific subpopulations of PCNSL patients to better identify those who could benefit from rituximab. 

Given that the effect of rituximab on survival endpoints in the treatment of PCNSL continues to be uncertain, clinical decision making should also be guided by data on treatment-related toxicity, neurocognitive functioning and quality-of-life [49,50,51]. Recently, these data on health-related quality of life (HRQoL) from the HOVON/ALLG-trial have been published [52]. In a follow-up period of 24 months after the completion of treatment, in 80% of the original study population, the addition of rituximab to standard chemotherapy did not impact the HRQoL. Lymphoma treatment as a whole resulted in clinically relevant improvements in functioning and well-being, as compared with baseline. The effects of whole-brain radiotherapy consolidation on neurocognitive functioning and HRQoL remain outside the scope of this article, but the authors surprisingly did not report a deterioration of HRQoL nor neurocognitive functioning in the first two years after 30 Gy radiotherapy in this cohort. 

## 5. Conclusions

In the treatment of PCNSL, there is still no consensus whether the addition of the monoclonal antibody rituximab to methotrexate-based chemotherapy improves outcomes. The meta-analysis of two randomized controlled trials shows, with low certainty, a possible beneficial effect on progression-free survival, but no such effect on overall survival. There appears to be no significant surplus toxicity or impact on HRQoL associated with the treatment and rituximab is generally well tolerated. For these reasons, despite the lack of definitive evidence, many PCNSL patients are already being treated with rituximab. Not implementing rituximab in your PCNSL-treatment, however, is still a defendable alternative. 

## Figures and Tables

**Table 1:** Overview of the randomized controlled trials on the effect of rituximab in primary central nervous system lymphoma (PCNSL).

Study Parameters	IELSG32	HOVON 105/ALLG
Study design	Multicenter randomized phase II	Multicenter randomized phase III
Recruitment period	2010–2014	2010–2016
Median age (years)	57	61
Sample size	227	200
Median follow-up (months)	30	32.9
Intervention group	MTX + cytarabine + rituximab	MBVP + rituximab
Control group	MTX + cytarabine	MBVP
Consolidation	WBRT or ASCT	Cytarabine and WBRT ^1^
Primary endpoint	CR 30% in rituximab	1y-EFS 52% in rituximab
	CR 23% in control	1y-EFS 49% in control
	HR 0.74, 95% CI 0.43–1.29, *p* = 0.29	HR 1.00, 95% CI 0.70–1.43, *p* = 0.99
Secondary endpoint	2y-PFS 46% in rituximab	2y-OS 71% in rituximab
	2y- PFS 36% in control	2y-OS 65% in control
	HR 0.52, 95% CI 0.32–0.86, *p* = 0.051	HR 0.93, 95% CI 0.59–1.44, *p* = 0.74
Conclusion	No beneficial effect of rituximab	No beneficial effect of rituximab

^1^ WBRT was only considered in patients younger than 61 years. MTX = methotrexate; MBVP = combination of methotrexate, carmustine, teniposide, prednisone; WBRT = whole-brain radiotherapy; ASCT = autologous hematopoietic stem cell transplantation; CR = complete remission rate; EFS = event-free survival; PFS = progression-free survival; OS = overall survival.

**Table 2:** Overview (non-exhaustive) of historical [29,30,31,32,33] and prospective [34,35,36] cohort analysis on the effect of rituximab in PCNSL.

Publication	Outcome in Experience without Rituximab	Outcome in Experience with Rituximab	Number of Patients in Cohort	Recruitment Period of Cohort
Kansara et al. [29]	MTX 5y-OS 38%	MTX + R 5y-OS 38%	74	2000–2013
Birnbaum et al. [30]	MTX + IFO median PFS 18 months	MTX + IFO + R median PFS 30 months	36	2007–2010
Holdhoff et al. [31]	MTX median PFS 5 months	MTX + R median PFS 27 months	81	1995–2012
Mocikova et al. [32]	MPV median PFS 10.9 months	MPV + R median PFS 22.9 months	164	2002–2012
Madle et al. [33]	Systemic treatment 3y-OS 40%	Systemic treatment + R 3y-OS 78%	81	2000–2011
Morris et al. [34]		R-MVP 3y-OS 87%	52	2002–2009
Shah et al. [35]		R-MVP 2y-OS 67%	30	2002–2005
Fritsch et al. [36]		R-MCP 3y-OS 31%	28	2005–2009

MTX = methotrexate; R = rituximab; OS = overall survival; IFO = ifosfamide; PFS = progression-free survival; MPV = methotrexate, procarbazine, vincristine; MCP = methotrexate, procarbazine, lomustine.
